# P25 Early onset congenital sarcoidosis: Challenges in the management of recurrent severe bilateral uveitis and inflammatory arthritis

**DOI:** 10.1093/rap/rkab068.024

**Published:** 2021-10-19

**Authors:** Salamat Ullah, Farhan Javed, Joel David, Gopinath Reddy, Ian Fearnley, Periyasamay Kumar, Rachel Jeffery

**Affiliations:** 1 Northampton General Hospital, Northampton, United Kingdom; 2 Oxford University Hospital, Oxford, United Kingdom; 3 University Hospitals of Leicester, Leicester, United Kingdom

## Abstract

**Case report - Introduction:**

We report a rare case of 20-year-old male diagnosed with congenital sarcoidosis (Blau Syndrome) presenting with a triad of skin rash, arthritis and bilateral uveitis.

He has had recurrent flares of sight-threatening bilateral uveitis, as well as persistent arthritis, which has continued despite treatment with multiple immunosuppressive agents and corticosteroids. The best control has been achieved with combination of methotrexate and infliximab which he has returned to; however, complications of frequent infections has necessitated breaks in treatment.

**Case report - Case description:**

A 4-year-old male presented with inflammatory skin rash which was biopsied and diagnosed as sarcoid lesion. He developed bilateral blurring of vision and diagnosed with uveitis, which was treated with topical and oral steroids. He then experienced multiple joint pains with synovitis. His father had been diagnosed from a young age with sarcoidosis. Other possible causes were ruled out.

From early childhood, our patient was treated with methotrexate, then methotrexate and azathioprine for recurrent uveitis, but required long-term oral prednisolone. He started infliximab with methotrexate in adolescence with good response and weaned his steroids. However, uveitis flared when infliximab was stopped for appendicectomy, requiring rescue with oral and intraocular steroids.

Further flares with cystoid macular oedema secondary to sarcoid uveitis and arthritis flares required high-dose steroids, contributing to iatrogenic cushingoid syndrome. Beta-haemolytic streptococcal infection led to guttate psoriasis, after which he had more persistent polyarticular inflammation and tenosynovitis. Antibodies were detected against infliximab although clinically uveitis continued to respond. Adalimumab caused site injection reactions. MDT discussions recommenced higher dose infliximab. Long-standing nausea after methotrexate, oral or subcutaneous, despite folic acid and anti-emetics, became intolerable. Transfer of care to another rheumatology centre focused on trying to control his arthritis. Leflunomide and then mycophenolate-mofetil replaced methotrexate. Infliximab was then switched to tofacitinib (developed severe headache) and then baricitinib. Joint symptoms improved on baricitinib, and nausea reduced with mycophenolate; however, his uveitis flared severely again requiring further high-dose oral and intraocular steroid rescue. Further MDT and patient discussion resulted in restarting methotrexate 20mg weekly (enduring nausea) and infliximab 5mg/kg monthly. Uveitis settled and arthritis control remained stable over the last 12 months with reduction in prednisolone to 5mg daily. Recurrent infections with recent pilonidal sinus continue to cause treatment interruptions.

**Case report - Discussion:**

Sarcoidosis is a chronic, idiopathic multisystem, granulomatous disorder rarely occurring in children. Early onset sarcoidosis/Blau syndrome (familial) is distinct, presenting in children around age 4 years and characterised by the triad of skin, joint and eye manifestations without the pulmonary involvement seen in adults. Children with Blau syndrome can develop severe complications such as blindness, growth retardation, heart involvement, renal failure and death.

Our patient was diagnosed with Blau syndrome based on the symptom triad and NOD2 gene mutation positive. He had recurrent flares of severe uveitis and difficult to manage joint disease involving his wrists/hands, shoulders, knees, ankles with tenosynovitis. He required shoulder decompression, knee-meniscal surgery and plastic-surgery to finger tendons. With early treatment he has grown well and preserved visual acuity (6/9 in both eyes), with no other organ involvement.

He works full-time. He has suffered from depression and struggled with body image issues, particularly from cushingoid changes. Difficult patient choices balanced medication side effects and control of eye/joint inflammation. He endures nausea after weekly methotrexate and some ongoing joint symptoms to enable his uveitis-responsive treatment to be prioritised.

Recurrent infections interrupted use of the most efficacious immunosuppressive therapy (infliximab and methotrexate). Jak Inhibitors provided some benefit for joint symptoms but did not control his uveitis. Different mechanisms seem to be important in the perpetuation of his uveitis and arthritis given the differential responses to treatment. Other treatment options such as anti-IL1 therapy were considered; however, limited data for eye inflammation results in patient continuing on his current therapy.

Multi-disciplinary care/co-ordination is essential in managing this complex, rare condition to balance optimisation of his medications to treat his most organ threatening and debilitating symptoms.

Genetic studies have enabled confirmation of the familial basis of his condition and autosomal dominant inheritance risk.

**Case report - Key learning points:**

1. Multidisciplinary team approach is crucial for the management of complex patients.
2. Patient-centred care in adolescents and young adults is of extreme importance considering their treatment choices and lifestyle along with their expectations and ongoing challenges in their daily life due to burden of disease and treatment side-effects.
3. Infliximab and methotrexate remained the best combination in controlling eye disease and joint disease activity in this case.
4. Early treatment with immunosuppressive therapy has modified potential growth retardation and organ damage in this condition.
5. Blau syndrome is a rare differential diagnosis in children presenting with juvenile-onset inflammatory arthritis and uveitis.
6. There is little evidence for the use of different immunosuppressive agents in the management of severe recurrent uveitis and inflammatory arthritis in this condition and optimising the balance of control of inflammatory features. Treatment options have been used from therapeutic regimens for juvenile idiopathic arthritis and adult inflammatory arthritis.

